# Unexpected plant odor responses in a moth pheromone system

**DOI:** 10.3389/fphys.2015.00148

**Published:** 2015-05-12

**Authors:** Angéla Rouyar, Nina Deisig, Fabienne Dupuy, Denis Limousin, Marie-Anne Wycke, Michel Renou, Sylvia Anton

**Affiliations:** ^1^Institut d'Ecologie et des Sciences de l'Environnement de Paris, INRA, Université Pierre et Marie CurieVersailles, France; ^2^Neuroéthologie-RCIM, INRA-Université d'AngersBeaucouzé, France

**Keywords:** insect olfaction, sex pheromone, volatile plant compounds, interaction, olfactory receptor neuron, antennal lobe, central neuron

## Abstract

Male moths rely on olfactory cues to find females for reproduction. Males also use volatile plant compounds (VPCs) to find food sources and might use host-plant odor cues to identify the habitat of calling females. Both the sex pheromone released by conspecific females and VPCs trigger well-described oriented flight behavior toward the odor source. Whereas detection and central processing of pheromones and VPCs have been thought for a long time to be highly separated from each other, recent studies have shown that interactions of both types of odors occur already early at the periphery of the olfactory pathway. Here we show that detection and early processing of VPCs and pheromone can overlap between the two sub-systems. Using complementary approaches, i.e., single-sensillum recording of olfactory receptor neurons, *in vivo* calcium imaging in the antennal lobe, intracellular recordings of neurons in the macroglomerular complex (MGC) and flight tracking in a wind tunnel, we show that some plant odorants alone, such as heptanal, activate the pheromone-specific pathway in male *Agrotis ipsilon* at peripheral and central levels. To our knowledge, this is the first report of a plant odorant with no chemical similarity to the molecular structure of the pheromone, acting as a partial agonist of a moth sex pheromone.

## Introduction

Most insects use olfactory cues to communicate and find resources necessary for survival and reproduction. Olfactory-guided behavior, as well as the detection and central processing of sex pheromone and general odor cues have been particularly well studied in moths, in which the olfactory system shows a prominent sexual dimorphism related to sex pheromone communication. Female moths release a species-specific sex pheromone blend, which triggers a well-described oriented flight behavior along the pheromone plume in males, leading them toward the pheromone source (Cardé and Willis, 2008). Both sexes use also flower odors to find nectar sources, and females use plant volatiles in their search for oviposition sites on host plants. Male moths might also use host-plant volatiles to approach the habitat from which females are likely to be calling (Light et al., 1993; Coracini et al., 2004).

Insects detect odorants with olfactory receptor neurons (ORNs), housed within cuticular sensilla on their antennae. In male moths, species-specific pheromones and volatile plant compounds (VPCs) are usually detected and processed by two distinct olfactory pathways (Masson and Mustaparta, 1990) and separation between pheromone and plant signals occurs already at the peripheral level. Information about the pheromone blend is transferred from the antennae via the axons of pheromone-specific olfactory receptor neurons (Phe-ORNs) to the primary olfactory center, the antennal lobe (AL) where it is processed in a male-specific area, the macroglomerular complex (MGC). Information about plant odors is transferred via a different class of olfactory receptor neurons (VPC-ORNs) and processed in sexually isomorphic areas of the AL, the ordinary glomeruli (OG) (Hansson and Anton, 2000). Because natural insect behavior results generally from the integration of multiple information sources, determining to which extent the two sub-systems are completely separated has recently become a very important case study in sensory ecology. More and more information is accumulating, indicating that simultaneous stimulation with pheromone and plant odors leads to interactions at all levels from detection up to behavioral output. The most frequently observed effect of mixture interaction in Phe-ORNs is suppression of pheromone responses when a VPC is added (Den Otter et al., 1978; Kaissling et al., 1989; Pophof and Van Der Goes Van Naters, 2002; Party et al., 2009; Rouyar et al., 2011). In the antennal lobe, plant volatiles either enhance pheromone responses (Namiki et al., 2008; Trona et al., 2010), or have a suppressive effect (Chaffiol et al., 2012; Deisig et al., 2012). Behavioral tests in the wind tunnel or in the field show often synergistic effects of plant odors added to the pheromone, more males being attracted to the mixture. In *Spodoptera exigua*, for example, phenyl-acetaldehyde, (Z)-3-hexenyl acetate or linalool increased captures of males in pheromone traps (Deng et al., 2004), and in wind tunnel experiments (Z)-3-hexen-1-ol, (+)-terpinen-4-ol, (E)-β-caryophyllene and methyl salicylate released with sub-optimal pheromone doses caused a synergistic effect in *Eupoecilia ambiguella* (Schmidt-Büsser et al., 2009). However, in spite of the observed interactions, so far the pheromone and plant odor inputs to the nervous system have been postulated to be highly separated up to their integration in the moth ALs. This consensual view of a high specificity of the pheromone sub-system arises from the repeated observation of a narrow chemical tuning of the pheromone receptor neurons to pheromone-like structures (Masson and Mustaparta, 1990). This high pheromone selectivity has been confirmed by heterologous expression of sex-pheromone receptors from several moth species, confirming they selectively bind the pheromone components over their close structural isomers (Nakagawa et al., 2005; Wanner et al., 2010; Liu et al., 2013). It is thus generally admitted that these olfactory receptors, narrowly tuned to pheromone components, act as molecular filters, preventing the activation of the pheromone pathway by general odorants. However, most studies have focused on pheromone-related compounds, so the capacity of general odorants to be bound to pheromone receptors should be more specifically addressed. As a matter of fact, if in *Helicoverpa zea* or *Spodoptera littoralis*, plant volatiles alone did not elicit responses from the Phe-ORNs (Ochieng et al., 2002; Party et al., 2009), high doses of plant compounds have been observed to activate Phe-ORNs in *Agrotis segetum* (Hansson et al., 1989). We revisit here the question of the sensitivity of the moth pheromone sub-system to plant odorants.

In the present study, using electrophysiological recordings and *in vivo* calcium imaging we show how plant volatiles in the noctuid moth *Agrotis ipsilon* activate not only the plant odor-specific pathway but also Phe-ORNs and the sex pheromone-specific MGC. The VPC heptanal used primarily in our study, with its seven-carbon chain length and an aldehyde function is emitted by various flowers such as linden flowers (*Tilia* sp.) that are attractive to *A. ipsilon* when searching for food (Wynne et al., 1991; Zhu et al., 1993), and is structurally different from the three acetates that constitute the sex pheromone blend of *A. ipsilon*. In the wind tunnel, male *A. ipsilon* were previously shown to be attracted by a linden flower extract (Deisig et al., 2012). We compare here in detail the upwind flight behavior toward heptanal and the pheromone blend. To determine if effects found for heptanal are specific, we also tested Phe-ORN responses to different other plant volatiles.

## Materials and methods

### Insects

Larvae of *A. ipsilon* were reared in the laboratory on an artificial diet in individual plastic containers at 23°C and 60% relative humidity until pupation. Sexes were separated at pupal stage, and females and males were kept in separate rooms under a reversed 16 h:8 h light:dark photoperiod under similar temperature and humidity conditions. Newly emerged adults were collected every day and provided *ad libitum* with a 20% sucrose solution. The day of emergence was considered day zero of adult life. Four or five day old sexually mature virgin males were used for electrophysiological, optical imaging and wind tunnel experiments. All experiments were performed during the scotophase, when male moths are sexually active. Some complementary experiments were run on males of *S. littoralis*, which were reared under the same conditions as *A. ipsilon*.

### Chemicals

#### Sex pheromones

We used a highly attractive synthetic sex pheromone blend of *A. ipsilon* based on the three components identified previously in natural extracts of the female gland (Picimbon et al., 1997; Gemeno and Haynes, 1998): (Z)-7-dodecen-1-yl acetate (Z7-12:OAc), (Z)-9-tetradecen-1-yl acetate (Z9-14:OAc) and (Z)-11-hexadecen-1-yl acetate (Z11-16:OAc), mixed at a ratio of 4:1:4. This blend was further proven to be the most attractive to males in field tests (Causse et al., 1988) and it has been shown to elicit the same behavior as natural extracts in a wind tunnel (Barrozo et al., 2010b; Vitecek et al., 2013). We preferred in this paper to use the pheromone as a whole to investigate heptanal interactions with the complete stimulus at all integration levels. The three compounds were purchased from Sigma Aldrich (Saint-Quentin Fallavier, France) and diluted in hexane (>98% purity, CAS 110-54-3, Carlo-Erba, Val-de-Reuil, France). Amounts of 10 ng and/or 100 ng of the sex pheromone blend were used in the electrophysiological and calcium imaging experiments; these doses had previously been described as behaviorally and electrophysiologically active (Gadenne et al., 2001; Barrozo et al., 2010a; Chaffiol et al., 2012; Deisig et al., 2012). For ORN recordings from pheromone sensilla in *S. littoralis*, the major sex pheromone component, (Z)-9 (E)-11 tetradecadienyl acetate (Z9,E11-14:Ac) was used (Ljungberg et al., 1993).

#### Volatile plant compounds

Heptanal (98% purity, CAS 66-25-1, confirmed by GC analysis, revealing no traces of pheromone compounds) and VPCs belonging to different chemical families (aldehydes, acetates, terpenes as well as one aromatic compound) were used for some experiments: (Z)-3-hexenyl acetate (Z3-6Ac) (98% purity, CAS 3681-71-8), hexanal (>99% purity, CAS 66-25-1), octanal (98% purity, CAS 124-30-0), linalool (97% purity, CAS 78-70-6), 2-phenylethanol (99% purity, CAS 60-12-8), and α-pinene (97% purity, CAS 7758-70-8). Mineral oil (CAS 8042-47-5) was used to prepare volume-to-volume dilutions at 0.1 and 1%. All compounds were purchased from Sigma Aldrich (Sigma Aldrich, Saint-Quentin Fallavier, France).

### Olfactory stimulation

Odorants were delivered as described previously (Rouyar et al., 2011). Briefly, charcoal-filtered air was re-humidified and divided in eight equal flows (220 ml/min) directed each to a three-way miniature valve. From there the flow could be directed to one 4 ml glass vial containing the stimulus source by activating the appropriate valve. The connections to the vials were made using PTFE tubing (1.32 mm ID) and hypodermic needles (18 G size). For practical reasons, due to their differences in volatility and polarity it was not possible to use the same type of stimulus sources for pheromone and heptanal or other volatile compounds. For VPCs, the vial contained 1 ml of solution in mineral oil at the appropriate concentration vol/vol. For the pheromone, the vial contained a section of PTFE tubing (1.6 mm ID; L = 20 mm) directly connected to a hypodermic needle and containing 10 or 100 ng of the sex pheromone. Stimulus- and clean air-carrying tubes were maintained together in a 10 cm long metal tubing constituting the stimulation pencil. A plastic cone of a P1000 pipette was placed at the output of the stimulation pencil to serve as a mixing chamber. It was placed approximately 5 mm in front of one of the moths' antennae and focused on antennal sensilla, when we recorded ORNs. In order to stimulate the whole antenna, the cone was placed 20 mm in front of the head in optical imaging experiments, or 5 mm in front of the antenna when we recorded MGC neurons intracellularly. Programming of the electric valves was performed using a Valve Bank (AutoMate Scientific, Berkeley, USA) synchronized with the PC acquisition software.

### Measures of aerial concentrations of VPCs

To trace the olfactory stimulus at the output of the delivery system we used a fast response miniature photo ionization detector (Justus et al., 2002) (PID, from Aurora Scientific Inc, Aurora, Canada). Pheromone components could not be traced by this technique due to the high ionization potential of the pheromone molecules, which is above the energy of the PID lamp (10.6 eV). In turn, as all VPCs except phenyl ethanol and octanal produced measurable PID signals in the relevant concentration range we could estimate their concentrations in ppm_V_ at the output of the stimulator used in electrophysiological experiments.

In a first step, we calibrated the response of the PID to a source of known increasing concentrations of the VPCs. To generate these concentrations, we used an automatic syringe driver (Harvard Apparatus, model 55-2222) equipped with a 250 μl gastight microsyringe (Hamilton) to inject the pure compound at known rate into a controlled flow of charcoal filtered air (60 l/h, controlled by a flowmeter Meterate tube). The probe of the PID was inserted into the flow and the PID gain was settled at x10. The amplitude of the PID signal was measured after each increase of the delivery rate. Knowing the air flow rate and the chemical injection rate, it is possible to calculate the theoretical concentration in the final air (Chaffiol, personal communication) according to Equation 1:

$$C=({\text{F}}_{\text{chem}}*(\mu /\text{M})*\text{Vmolar})/{\text{F}}_{air}$$

Where:

C = final concentration of the compound in ppb_V_

F*_chem_* = flow rate of the chemical (μl/h)

F*_air_* = (m^3^/h)

μ = density of the compound (g/cm^3^)

M = molecular weight of the compound (g/mol)

Vmolar = molar volume for ideal gasses at 25°C (25.10^3^ ml).

The speed of the syringe driver was adjusted to the suitable rate, and the concentration was allowed to stabilize for 1 min after which the output signal of the PID was measured three times. Subsequently the speed of the syringe pump was increased to reach the next rate step. Measures were done for at least 10 different rates, presented in increasing orders, until the saturation response of the PID (10 mV) was reached. The rates were converted (Equation 1) into ppm_V_ and the experimental data were fitted to a polynomial regression according to the procedure recommended by the PID constructor for calibration (Equation 2):

$$\left({\text{S}}_{\text{PID}}={\text{aC}}^{\text{2}}+\text{bC}\right)$$

where S_PID_ is the amplitude of the PID response in volts, and C the concentration in ppm_V_.

For measurements, the probe of the PID was introduced into the olfactory stimulator to quantify the concentrations of the compounds in the odorized air flows. Compounds were delivered at three dilution levels (0.5, 1, and 10% vol/vol in mineral oil) in the same conditions used for our electrophysiological experiments, and measures were repeated five times. The data in mV were converted into ppm_V_ using equation and values for 0.1% extrapolated from the resulting curve. The concentration measures are summarized in Table 1 where data for 0.1% were extrapolated.

**Table 1:** **Estimation of the concentrations in ppm_V_ at the output of the stimulator used in the electrophysiological experiments for five different volatile plant compounds released from sources containing 1 ml of mineral oil with 0.1, 1, and 10% of the respective compound as calculated from measurements with a photo ionization detector**.

**Compound**	**0.1% v/v**	**1% v/v**	**10% v/v**
Heptanal	3.9	14.4	119.1
α-pinene	0.6	0.7	8.0
Linalool	0.7	3.1	14.4
Hexanal	16.5	19.4	95.6
Z3-6Ac	0.7	0.9	17.2

### Electrophysiology

#### Single sensillum recording of ORNs

Males were briefly anesthetized with CO_2_ and restrained in a Styrofoam holder. One antenna was immobilized with adhesive tape. Single sensillum recordings were performed with electrolytically sharpened tungsten wires. The reference electrode was inserted into the antenna, 1-3 segments from the segment carrying recorded sensilla, and the recording electrode was inserted into the base of a sensillum. We recorded two types of sensilla: long trichoid hairs based on antennal branches known to house Phe-ORNs and short trichoid hairs situated on the antennal stem known to house VPC-ORNs in a closely related species, *A. segetum* (Hansson et al., 1989). Recording and reference electrodes were connected to a Neurolog preamplifier (Digitimer, Hertfordshire, UK). The signal was filtered (0.2–10 kHz) and amplified 1000 times. The electrophysiological activity was sampled at 10 kHz and 12 bit resolution with a Data Translation DT3001 analog to digital card. Signals were monitored on the computer screen using Awave software (Marion-Poll, 1995). For analysis, spike sorting and extraction of spike occurrence times from the recordings were also done using Awave software. In some recordings from long trichoid hairs housing Phe-ORNs, the activities of two neurons with different spike amplitudes were analyzed, but only one neuron showed changes in firing rate in response to the sex pheromone. Also earlier recordings from Phe-ORNs showed that several neurons could be present in a given sensillum in *A. ipsilon*, but in all cases only one of them responded to a pheromone compound (Renou et al., 1996; Jarriault et al., 2010).

#### Intracellular recordings of MGC neurons

Males were slipped inside a 1 ml plastic pipette cone cut at the top. Only the head exceeded the plastic cone and was fixed with dental wax to prevent any movement. As described earlier (Gadenne and Anton, 2000), the head capsule was opened and tracheal sacs and muscles were removed from the front of the head to expose the brain. The neurolemma was removed from the surface of the antennal lobe to facilitate microelectrode penetration. Standard intracellular recording techniques were used (Christensen and Hildebrand, 1987). The preparation was superfused with Tucson Ringer (Christensen and Hildebrand, 1987). The microelectrode was randomly placed into the MGC. Electrode resistances were about 20–100 MΩ. The reference electrode was placed in contact with the brain. Signals were amplified with an AxoClamp-2B amplifier (Molecular Devices, Sunnyvale, California, USA). Neural activity was recorded, digitized, and spike occurrence times extracted using P-clamp software (Molecular Devices, Sunnyvale, California, USA).

#### Experimental protocol

#### Phe-ORN and MGC neuron responses to heptanal

We tested the response of Phe-ORNs and MGC neurons to heptanal by stimulating the antenna with a 200 ms heptanal puff at a dose of 0.1 and 1%. Phe-ORNs were recorded during 1 min and the odorant stimulation started at 30 s, lasting for 200 ms. For MGC neurons, odorant stimulation started 5 s after recording onset and inter-stimulus-intervals lasted for 10 s. Ten second interstimulus intervals are sufficient to allow AL neurons to reach the pre-stimulus spontaneous activity level and have been used in earlier studies of AL neurons in *A. ipsilon* (e.g., Barrozo et al., 2010a). We tested the pheromone at 100 ng and as controls, pure mineral oil and hexane, each for 200 ms.

#### Phe-ORN responses to other VPCs

As we obtained unexpected responses of Phe-ORNs to heptanal, we also tested the effects of other VPCs on these ORNs: Z3-6Ac, hexanal, octanal, linalool, 2-phenylethanol and α-pinene. To check if we were recording from Phe-ORNs, we first stimulated the antenna with a 100 ng pheromone puff. Then puffs of the other compounds at 1% were randomly presented. As controls, the solvents hexane and mineral oil were tested.

#### VPC-ORN responses to pheromone

To check if VPC-ORNs also respond to the pheromone, we stimulated short trichoid sensilla situated on the stem of the antenna with 100 ng pheromone during 200 ms. To test if we had indeed contact with VPC-ORNs, we presented puffs of 0.1% of the VPCs heptanal, Z3-6Ac, hexanal, octanal, linalool, 2-phenylethanol, and α-pinene. As controls, we tested the solvents hexane and mineral oil.

#### Species specificity of Phe-ORN responses to VPCs

To test if the effect induced in Phe-ORNs by VPCs is specific to *A. ipsilon*, we recorded long trichoid sensilla in male *S. littoralis*, which have been shown to house one Phe-ORN tuned to the major sex pheromone compound Z9,E11-14:Ac. We stimulated sensilla with heptanal and linalool at 1% during 200 ms. To test if we were recording from Phe-ORNs, we first stimulated the antenna with 100 ng pheromone during 200 ms. As controls we presented the two solvents hexane and mineral oil.

### Calcium imaging

#### Insect preparation

Males were mounted individually in Plexiglas chambers and the head was fixed to prevent movements. The head capsule was opened and glands and trachea were removed. Ten microliter of dye solution (50 mg Calcium Green 2-AM dissolved in 50 ml Pluronic F-127, 20% in dimethylsulfoxide, Molecular Probes, Eugene, OR, USA) were bath-applied on the brain for a minimum of 1 h. The brain was then washed with saline solution (Tucson Ringer) containing 150 mmol/l NaCl, 3 mmol/l CaCl_2_, 3 mmol/l KCl, 10 mmol/l N-Tris-methyl-2-aminoethanesulfonic acid buffer, and 25 mmol/l sucrose (pH 6.9).

#### Data acquisition

Recordings were done using a T.I.L.L. Photonics imaging system (Martinsried, Germany) coupled to an epifluorescent microscope (Olympus BX-51WI, Olympus, Hamburg, Germany) equipped with a 10x water immersion objective. Images were taken using a 1004 × 1002 pixel 14-bit monochrome CCD camera (Andor iXON) cooled to −70°C. Each measurement consisted of 80 frames at a rate of 5 frames/s (integration time for each frame: 10–15 ms). The excitation light was applied using a monochromator (T.I.L.L. Polychrom V). The microscope was equipped with a GFP-BP filter set composed of a 490 nm dichroic beamsplitter and a 525/550 nm emission filter.

#### Data analyses

Because identification of individual glomeruli by anatomical staining of the AL after calcium imaging experiments is not possible in *A. ipsilon*, we defined regions of interests (ROI), possibly referring to individual glomeruli for OGs. Homologous ROIs could be identified by superposing activity maps using Adobe Photoshop (CS3). Raw data were analyzed using custom-made software written in IDL (Research Systems Inc., Colorado, USA) and Visual Basic (Microsoft Excel). Each recording corresponded to a three-dimensional matrix with two spatial dimensions (x and y size in pixels of the ROI) and a temporal dimension (length of the recording, 80 frames). Signals were subjected to three treatments: (i) For reduction of photon (shot) noise, raw data were filtered in both the spatial and temporal dimensions using a median filter with a size of 3 pixels. (ii) Relative fluorescence changes (ΔF/F) were calculated as (F-F_0)_/F_0_, taking as reference background F_0_ the average of five frames (frames 5 to 10) before odor stimulation. (iii) To correct for bleaching and for possible irregularities of lamp illumination in the temporal dimension, we subtracted from each pixel in each frame the median value of all the pixels of that frame. The maximum signal was obtained about 3 s after odor onset (around frame 30) and the minimum about 12 s after odor onset (around frame 60). We present activity maps with the best possible spatial definition of odor-induced signals from frames 30 to 60 where each pixel represents the mean of its values at frames 29–31 minus the mean of its values at frames 59–61.

For quantitative analysis of the data, we focused on the fast (positive) signal component evoked by odor stimulations, which is related to an intracellular calcium increase from the extracellular medium, thought to reflect mostly pre-synaptic neuronal activity from ORNs (Galizia et al., 1998; Sachse and Galizia, 2003). For each identified activity spot, the time course of relative fluorescence changes was calculated by averaging 25 pixels (5 × 5) at the center of each activity spot and well within its borders. The amplitude of odor-induced responses was calculated as the mean of three frames at the signal's maximum (frames 29–31) minus the mean of three frames before the stimulus (frames 7–9). This value was then used in all computations.

#### Experimental protocol

Each animal was subjected to three series of olfactory stimulations with interstimulus intervals (ISIs) of 100 s. Odor stimulation started 3 s after recording onset and lasted for 200 ms. One AL was recorded in each insect. All animals were tested with a dose of 1% heptanal, 1% linalool as well as 100 ng of the pheromone. As control, we tested pure mineral oil and hexane.

### Wind tunnel experiments

The behavior of male moths responding to pheromone or heptanal was observed in a wind tunnel made of 19 mm thick Plexiglas, with a flight section of 190 cm length × 75 cm width × 75 cm height (VT Plastics, Genevilliers, France). Plexiglas doors on the front side of the tunnel allowed access to the test section. The down- and upwind ends were enclosed with screen made of white synthetic fabric to prevent the insects from escaping but let the air pass through. An exhaust fan at the downwind end of the tunnel sucked the air into the tunnel at a speed of 0.3 ms^−1^ and evacuated contaminated air to the outside of the building. The room housing the tunnel was maintained in darkness with a single red bulb to provide low intensity light for visual observations. Side infrared illumination for video tracking was provided by an array of eight 5 W IR lamps, of 54 LEDs each, emitting at 850 nm. A vertical screen bearing a randomly arranged pattern of 10 cm diameter black circles was positioned 30 cm behind the rear wall of the tunnel to provide visual cues to the moths outside the camera field.

Moth flight tracks were recorded and analyzed using Trackit 3D 2.0 (SciTracks, Pfaffhausen, Switzerland). Two cameras (Basler Pilot, piA640-210 m with Tamron ½” 4-12F/1.2 lenses) were positioned above the tunnel at 60 cm from each other to cover the whole flight section with overlapping fields. The images from the two cameras were analyzed in real time and the x, y, and z coordinates of each moth's position were extracted every 10 ms. Tracks were saved on the computer in form of “.csv” files that were further processed using scripts developed in R Core Team (2013).

Experiments were performed at 23°C, 40 ± 10% relative humidity, during the second half of the scotophase (i.e., 4–7 h after lights turned off) which corresponds to the peak activity of male *A. ipsilon*. Five-day old virgin males were tested. A single male was introduced inside a cage on a 36 cm high holder in the middle of the tunnel width and 160 cm downwind from the odor source. After allowing the moth to adapt to the airflow, we applied the odor stimulation and monitored the male's behavior for 3 min. We compared the responses of males to either the pheromone at 100 ng or heptanal at 0.1 or 1% dilutions. Control experiments (no odor) were performed with a clean filter paper as source. Each individual was tested only once. Olfactory stimuli were delivered using the same model of stimulator as in our electrophysiological experiments enabling to deliver odorants by switching solenoid-driven Lee micro valves via a Valve-Bank controller, with separate channels for each odorant. Hypodermic 18 G needles fixed in the middle of the upwind side of the tunnel were used as odor nozzles delivering odorized air flows at the upwind end of the tunnel. The solution of sex pheromone in hexane was deposited on a filter paper introduced in a 4 ml glass vial after solvent evaporation. Heptanal was diluted in 1 ml of mineral oil.

Four behavioral items were scored: activation (walking activity and/or wing fanning on the take-off platform), take off (taking off from the platform) partial flight during the test (flight half way between the release site and the odor source) and source flight (flight ending within 20 cm of the source) before the end of the test (180 s). All males stimulated with the pheromone blend showed activation and performed a take-off in less than 90 s after the beginning of the test, so 90 s was taken as the time limit for scoring these two items for all subsequent experiments.

To compare the orientation of males toward the wind direction in presence of the different odor sources the.csv files produced by Trackit were used to calculate distribution plots of the positions of the male along the tunnel width. The tracks were first smoothed using a local polynomial regression fitting [function “loess()” from R package “stats,” (R Core Team, 2013)]. We then extracted the section of the smoothed tracks from the departing point (platform), up to the point where the moth reached its maximum value on the length axis. Finally we calculated the cumulated distribution along the width axis of the males, within the whole length after take off (the first 10 cm from the platform were excluded) and plotted it for each treatment (pheromone, 1% heptanal, 0.1% heptanal, and control).

### Statistical analyses

For electrophysiological experiments, spike occurrence times were analyzed using custom-written R scripts (R Core Team, 2013). Firing rates were calculated using the local slope of the cumulative function of spike times (Blejec, 2005). The slope was calculated over a moving spike window between the n–2 and n+2 spikes (5 spikes). Thus, each spike was attributed a firing rate and its occurrence time. The maximum firing rate during the 1st second from stimulus start was measured for each recording. The mean ± standard error of the maximum firing rates was calculated for each stimulation. Data were compared using a Student *t*-test for paired data followed by tests to check for data set normality (Shapiro test) and variance homogeneity (Fisher-Snedecor test), concerning data from ORN recordings, or were compared using a Wilcoxon test for paired data for data from MGC neuron recordings.

The experimental decline of the averaged responses was fitted with an exponential asymptotic decay function by determining the non-linear least-squares estimates of parameter of an exponential model (function nls of R). Curves of firing rates were standardized relatively to the maximum firing rate. The asymptotic decay functions were estimated from the time of the maximum firing rate to 1 s after the stimulation times (Equation 3):

$$\text{FR}=\text{a}+{\text{b}}_{*}{\text{e}}^{\left(-\text{c }*\text{ time}\right)}$$

where *FR* is the maximum firing rate, a is the offset, b the initial firing rate, and c the rate coefficient of the curve. The time values for 90% decay (td90) were calculated from Equation 3.

We estimated the response latency for each recording using custom-written R scripts. First, we calculated a threshold for excitation response as the 95th percentile of spike firing rates before stimulation onset (spontaneous activity). Second, we looked for the first spike crossing this threshold within the expected response time window corresponding to 1 s after stimulation start. We defined this spike occurrence time as response latency. We compared median latencies between two treatments using a chi-square test.

Calcium responses induced by different odors in different glomeruli were compared using Statistica (Version'99, www.statsoft.com). We performed One- or Two-Way ANOVAs for repeated measures with the two factors odor and glomerulus. When interactions among factors were significant, simple effects were analyzed by means of a One-Way ANOVA with or without the RM factor, and then followed by a Tukey's test for *post-hoc* comparisons if necessary.

For wind tunnel experiments, a Fisher's exact test was used to compare scores of response of male moths to heptanal and the pheromone.

## Results

### Heptanal activates VPC-ORNs but also Phe-ORNs

**Figure 1:**
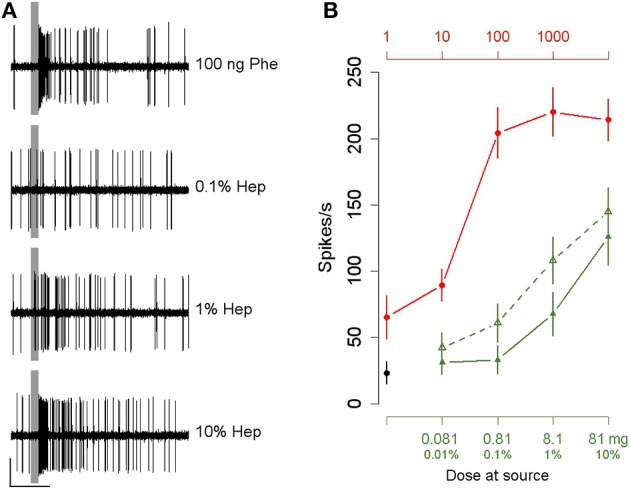
***Agrotis ipsilon* Phe-ORNs respond to heptanal. (A)** Typical examples of the responses of Phe-ORNs to the pheromone (Phe) and three doses of heptanal (Hep). Scale: vertical bar = 1 mV; horizontal bar = 1 s. The vertical gray bars indicate the stimulus (200 ms). **(B)** Dose-response curves of Phe-ORNs to a 200 ms puff of the pheromone or of Phe-ORNs and VPC-ORNs to heptanal. Mean of the maximum firing rates during the 1st second from stimulus start (± SEM). Doses of heptanal are in v/V % of dilution in mineral oil with their equivalance in mg (lower horizontal axis); doses of pheromone are in ng deposited on filter paper (upper horizontal axis). Phe-ORNs responded already to the lowest pheromone dose tested and the maximum firing rate increased with increasing doses (solid red line, *n* = 16). Phe-ORNs started to respond to heptanal at a dose of 1% (solid green curve, *n* = 12) while VPC-ORNs responded already at a dose of 0.1% (dashed green curve, *n* = 14). Controls (black dot) refer to pooled data of stimulation with pure hexane and pure mineral oil.

The antennae of *A. ipsilon* are bipectinate with ORNs tuned to pheromone (Phe-ORNs) mainly housed in the trichoid sensilla situated on the branches while ORNs tuned to volatile plant compounds (VPC-ORNs) are predominantly housed within sensilla localized on the antenna stem (Renou et al., 1996). We thus recorded from olfactory sensilla sampled either from the antennal branches or antennal stem and attributed functional types to ORNs according to the most active stimulus. An extensive screening of noctuid pheromone components has evidenced a majority (32 out 52) of Phe-ORNs responding exclusively to Z7-12:Ac, some neurons responding mainly to Z5-10:Ac but also to Z8 and Z9-12:Ac, and only one neuron responding to Z9-14:Ac, but no neuron responding to Z11-16:Ac (Renou et al., 1996). These functional types of Phe-ORNs were never encountered in a same sensillum. On the antennal branches, 92% of all Phe-ORNs encountered responded to Z7-12:Ac (Renou et al., 1996). Thus, we expected the latter neuron type to largely dominate our results. Our single-sensillum recordings showed that Phe-ORNs on the branches responded to the pheromone in a phasic-tonic mode (Figure 1A) and already at a dose of 1 ng (Figure 1B, red curve). Interestingly, these Phe-ORNs responded also to 1% heptanal (Figure 1B, solid green curve). This dilution corresponds to a total amount of 8.1 mg heptanal at the source and an aerial concentration of 14 ppm. The response amplitude to heptanal increased with increasing doses at the source but did not reach saturation at the highest dose tested (Figure 1B, solid green curve). The heptanal dose-response curve was clearly shifted toward higher concentrations, compared to the dose-response curve for the pheromone, indicating a lower potency for heptanal to activate Phe-ORNs.

On the other hand, the ORNs housed in olfactory hairs located on the antennal stem did not respond to the pheromone as expected from VPC-ORNs, but responded to heptanal with higher firing rates and a lower threshold compared to Phe-ORNs. These VPC-ORNs started to respond already at a dose of 0.1% corresponding to 0.81 mg (3.9 ppm_v_) heptanal at the source (Figure 1B, dashed green curve). In the following experiments, 10 and 100 ng or 0.1 and 1% will designate low and medium stimulus strengths for pheromone and heptanal, respectively.

**Figure 2:**
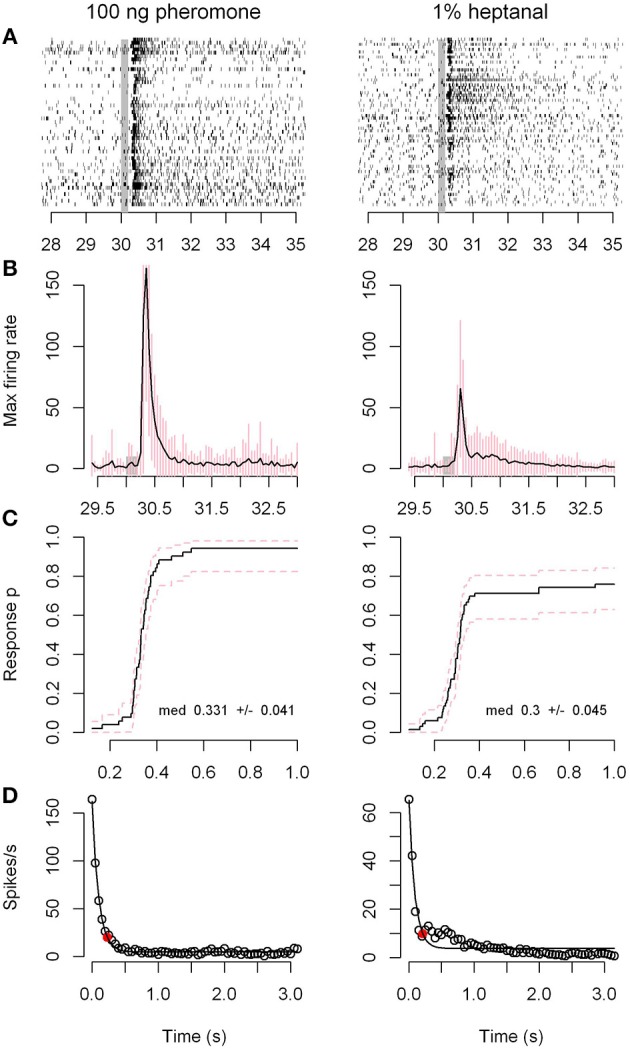
**Response dynamics of Phe-ORNs to the pheromone or heptanal are very similar**. The dynamics of the response of ORNs sampled on antennal branches to a 200 ms pulse of 100 ng pheromone (left column) or 1% heptanal (right column) are compared. **(A)** Raster plots of the firing activity of 51 individual neurons. The vertical gray bars show the stimulus time. **(B)** Frequency plots of the maximum firing rates for the same sample of neurons (time bin = 50 ms). Means of the 51 recordings. Error bars in pink represent standard deviation. **(C)** Kaplan-Meier curves of the response latencies; p is the proportion of neurons that responded to the olfactory stimulus at a given time. **(D)** Exponential decrease model for response end. The red dots represent the estimated values for 90% decrease.

We then compared the response dynamics of Phe-ORNs to the pheromone and 1% heptanal (Figure 2). All 51 Phe-ORNs examined responded to the pheromone by a simple phasic-tonic excitatory response (Figures 2A,B, left) with 0.331 s median latency (Figure 2C left). Among these 51 Phe-ORNS, 40 responded also to 0.1% heptanal, even though with lower firing frequencies (Figures 2A,B, right). The latency of the response to heptanal (median = 0.3 s Figure 2C right) was not significantly different from the response to pheromone (Student's *t*-test, *p* = 0.22). The firing responses to heptanal were generally phasic-tonic. However, the response patterns were more variable compared to those to pheromone (Figure 2A). In seven Phe-ORNs the responses to heptanal showed prolonged after-response firing activity, while in several others, responses presented a post-stimulus period of silence. Nevertheless, the decay of the response to pheromone or heptanal showed globally equivalent kinetics (Figure 2D), the firing rate decreased by 90% after 0.250 s with pheromone vs. 0.229 s in response to heptanal. However, the experimental data for heptanal were less well fitted to the theoretical exponential decay function than with the pheromone (Figure 2D), due to the post stimulus firing activity above the level expected from the simple exponential decay model in some neurons.

### Different other volatile plant compounds activate Phe-ORNs

**Figure 3:**
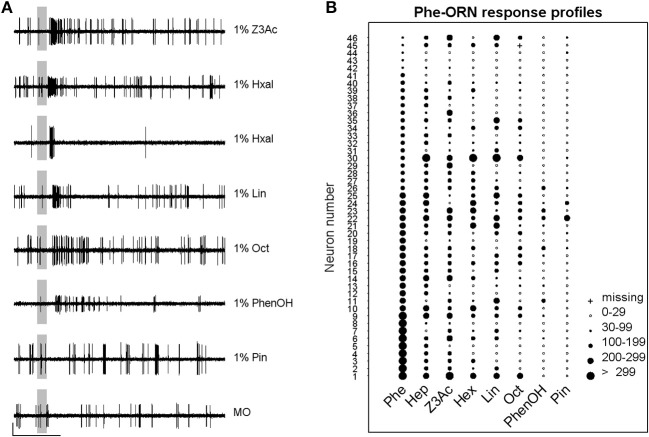
**Phe-ORNs respond to different volatile plant compounds. (A)** Typical examples of responses of Phe-ORNs to different volatile plant compounds. Single sensillum recordings from different sensilla show that Phe-ORN responses are similar between odors except for hexanal which induced two different response patterns. Scale: vertical bar = 1 mV; horizontal bar = 1 s. The vertical gray bar indicates the stimulus (200 ms). **(B)** Response-profiles of individual Phe-ORNs to seven VPCs. Each line presents the responses of a single ORN; each column shows the responses of different neurons to one odorant. The diameter of circles is proportional to the intensity of the response measured as the absolute maximum firing rate reached within 1 s after stimulus onset. Phe, pheromone; Hep, heptanal; Z3Ac, (Z)-3-Hexenyl acetate; Hxal, hexanal; Lin, linalool; Oct, octanal; PhenOH, 2-phenylethanol; Pin, α-pinene.

Most of the ORNs housed in the sensilla sampled on the branches also increased their firing in response to some of the VPCs tested at 1%, although the maximum firing rate in response to VPCs was generally lower compared to pheromone. Heptanal and the six additional VPCs elicited generally a single excitatory phase in Phe-ORNs (Figure 3A). Hexanal, however, triggered also an excitatory-inhibitory response in some Phe-ORNs (Figure 3A). Out of 46 tested ORNs situated on the branches of the antennae and showing clear responses to the pheromone, 40 responded also to Z3-6Ac, 27 to hexanal, 30 to linalool, 26 to octanal, 11 to 2-phenylethanol and only 2 to α-pinene (Figure 3B). However, the Phe-ORNs could not be sorted into functional sub-types according to their response profiles to the seven VPCs (Figure 3B). The intensity or frequency of the firing response of Phe-ORNs to VPCs was apparently not correlated to their chemical structure, nor to their volatility. For instance, aldehydes were not globally more active than the other VPCs; α-pinene was practically inactive although its vapor pressure is quite close to that of heptanal (500 and 300 Pa, respectively). For five Phe-ORNs, maximum firing rates were slightly higher for certain VPCs than for the pheromone itself.

### The pheromone does not activate ORNs on the antennal stem

**Figure 4:**
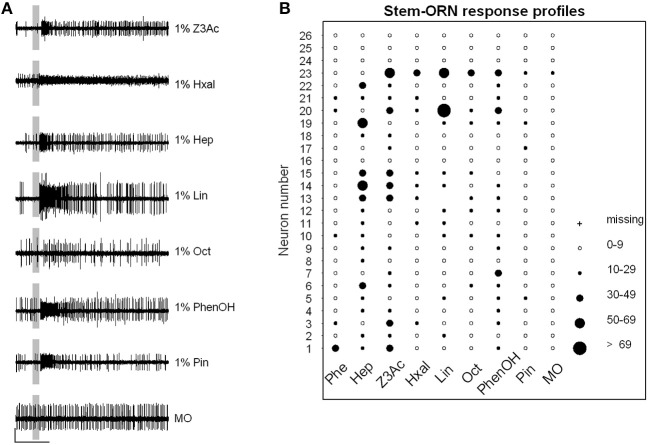
**VPCs activate general odorant ORNs. (A)** Typical examples of responses of Stem-ORNs from different sensilla to the set of VPCs. Single sensillum recordings show the presence of several spike sizes in most sensilla and generally excitatory responses in one of the ORNs of each sensillum. Scale: vertical bar = 1 mV; horizontal bar = 1 s. The vertical gray bar indicates the stimulus (200 ms). **(B)** Response-profiles of ORNs sampled on the antennal stem to seven VPCs and the pheromone. Only one of the ORNs responded to the sex pheromone. Each line presents the responses of a single ORN (*n* = 26); each column shows the responses of the different neurons to one odorant. The diameter of circles is proportional to the intensity of the response quantified as the absolute maximum firing rate reached during 1 s following stimulus onset. Phe, pheromone; Hep, heptanal; Z3Ac, (Z)-3-Hexenyl acetate; Hxal, hexanal; Lin, linalool; Oct, octanal; PhenOH, 2-phenylethanol; Pin, α-pinene; MO, mineral oil control.

Another set of single sensillum recordings was performed from the short sensilla trichodea localized on the antennal stem. The results revealed that only one of the sampled presumed VPC-ORNs (*n* = 26), which responded to at least one of the VPCs (examples of recordings in Figure 4A), responded also strongly to 100 ng of the pheromone (Figure 4B). This confirms a clear, but not exclusive spatial segregation of general odorant and pheromone detection in the antennae, most of the ORNs contained in the stem area being tuned to general odorants, while Phe-ORNs are mostly housed in branch hairs. The level of firing activity during responses was generally lower and stem-ORNs showed more specificity in their responses to the different VPCs compared to Phe-ORNs which each responded to several VPCs (Figures 3B, 4B).

### Heptanal does not activate Phe-ORNs of *S. littoralis*

**Figure 5:**
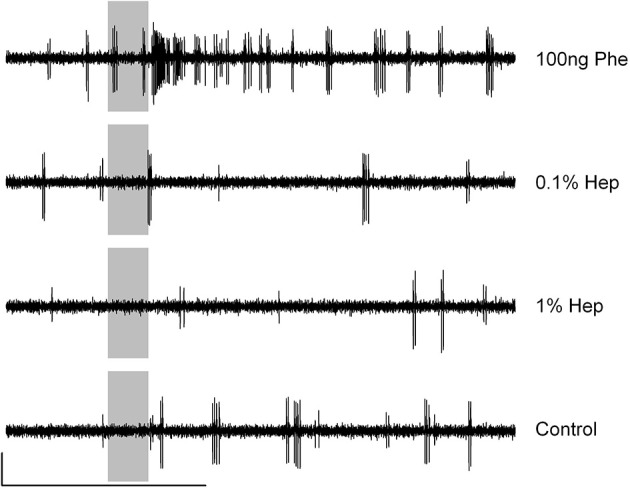
**Typical recordings of the firing activity of *Spodoptera littoralis* Phe-ORNs stimulated with pheromone or VPCs**. ORNs identified as pheromone receptor neurons from their position within long trichoid sensilla of the male antenna and their strong response to 100 ng Z9,E11-14:Ac (upper recording) did neither respond to 0.1% heptanal (middle trace) nor to 1% heptanal (lower trace). Scale: vertical bar = 1 mV; horizontal bar = 1 s. The vertical gray bar indicates the stimulus (200 ms).

To verify if VPC responses in Phe-ORNs are species-, or pheromone structure-dependent, we also recorded from 10 sensilla housing Phe-ORNs in another noctuid moth species, *S. littoralis*. Phe-ORNs responded to 100 ng of the major sex pheromone compound Z9,E11-14:Ac with a maximum firing rate of 228.1 spikes/s ± 30.6 (mean of 10 replicates ± SEM) while the firing activity in response to 1% heptanal (31.8 ± 16.3) was not significantly different from that to the control (11.3 ± 5.04; paired Student'*t*-test *p* = 0.148) (Figure 5).

### Heptanal evokes calcium responses in the MGC

**Figure 6:**
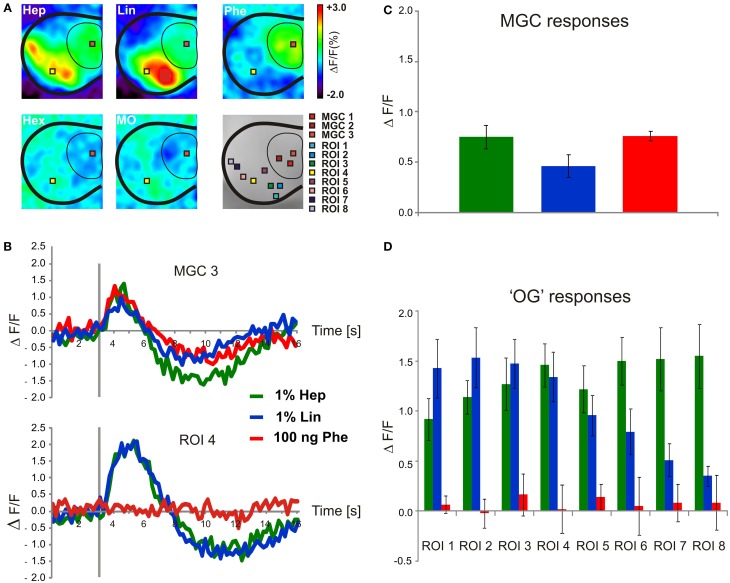
**Heptanal and pheromone evoke calcium responses in the AL. (A)** Odor induced *in vivo*-calcium imaging signals obtained in a male stimulated with heptanal, linalool, pheromone, pure hexane, and pure mineral oil. All maps are scaled to the same minimum/maximum. Eight ROIs were defined to cover the areas of OG (ROI1 to ROI8). Due to the large size of the MGC, three ROIs were defined within the MGC (MGC1 to MGC3). **(B)** Time course of odor-evoked calcium activity in the MGC and OG4 in the isomorphic glomeruli indicated in **(A)**. **(C)** Mean (± SEM) calcium response intensity in the MGC and **(D)** in OG1 to OG8 induced by heptanal (*n* = 6), linalool (*n* = 6), and the pheromone (*n* = 6), hexane (*n* = 4) and mineral oil (*n* = 4). The gray bar in **(B)** indicates the stimulus (200 ms). Hep, Heptanal; Lin, linalool; Phe, pheromone; MO, mineral oil; Hex, hexane; OG, ordinary glomeruli.

*In vivo* calcium imaging was performed to obtain a global pattern of the odor-evoked input to the antennal lobe. Global brain staining with a calcium-sensitive dye reveals odor-induced activity of all neuronal populations, however, due to their quantitative predominance, activity recorded originates mainly from ORN responses. Thus, such global responses are complementary to individual neuronal responses obtained with SSR or intracellular recordings. Stimulating the antennae of male *A. ipsilon* revealed calcium responses induced by the plant odors heptanal and linalool in the area of ordinary glomeruli. The pheromone elicited responses in the MGC. The solvents hexane and mineral oil did not elicit any response (Figure 6A). Odor-evoked signals were typical stereotyped biphasic signals usually obtained with bath application of the dye Calcium Green 2-AM, with first a fast fluorescence increase followed by a slow fluorescence decrease below baseline (Figure 6B; Galizia et al., 1997; Stetter et al., 2001; Sandoz et al., 2003). The two VPCs (heptanal and linalool) induced calcium responses in ROIs within the area of OGs (Figures 6B,D). Response intensity to 1% heptanal in ROIs 6, 7, and 8 was significantly stronger compared to response intensities induced by 1% linalool (Figure 6D, *Post-hoc* Tukey's test, ROI 6: *p* = 0.05; ROI 7: *p* = 0.02; ROI 8: *p* = 0.005), while linalool did not induce significantly stronger responses in any of the observed ROIs compared to heptanal (Figures 6B,D). The pheromone did not induce significant responses in the area of ordinary glomeruli (Figure 6D). In agreement with data obtained from our single sensillum recordings, not only stimulations with 100 ng sex pheromone, but also with 1% heptanal and 1% linalool evoked calcium responses in the pheromone-specific MGC of the AL (Figures 6B,C). Statistical analysis of the activity of 3 ROIs within the MGC area revealed that overall response intensity was not different between the 3 ROIs. Pooled data of the 3 ROIs within the MGC were not significantly different between heptanal-, linalool-, and pheromone-induced calcium signals [One-Way ANOVA, *F*_(2, 15)_ = 2.53, *p* = 0.11, Figure 6C, *n* = 6].

### Heptanal activates MGC neurons in the antennal lobe

**Figure 7:**
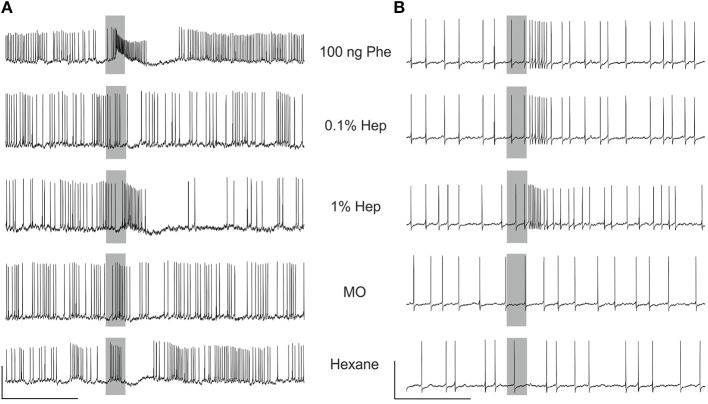
**MGC neurons respond to the pheromone and heptanal**. Examples of recordings of a biphasic **(A)** and a monophasic **(B)** neuron, responding similarly to the pheromone and heptanal. Note the responses in A to solvent controls, probably of mechanosensory origin (Jarriault et al., 2009). The gray bars indicate the stimulus (200 ms). Vertical scale A: 10 mV, B: 20 mV; horizontal scale 1 s.

**Figure 8:**
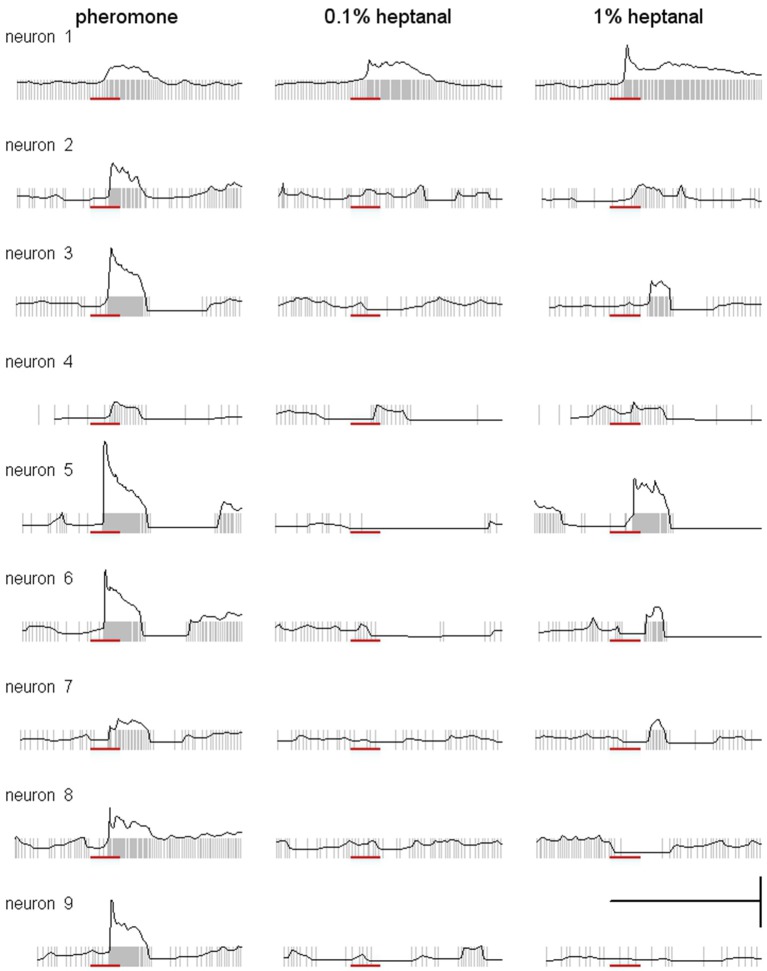
**Heptanal evokes more variable responses in MGC neurons than the pheromone**. Superimposed raster and frequency plots of responses of MGC neurons illustrating the variability of responses to heptanal between neurons and between pheromone and the two concentrations of heptanal within neurons. Scale: vertical bar = 100 spikes, horizontal bar = 1 s. Red bar indicates the stimulus (200 ms).

We recorded intracellularly from 35 MGC neurons with clear responses to the pheromone. Twenty-five of the recorded neurons showed an excitatory response followed by an inhibitory phase to 100 ng pheromone (type A neurons, Figure 7A) and 10 neurons showed a purely excitatory response (type B neurons, Figure 7B). Although more than half of the neurons responded to 1% heptanal with the same pattern as to the pheromone (Figure 7, and neurons 1 to 5 in Figure 8), responses to heptanal were more variable than to the pheromone (Figure 8). For both concentrations of heptanal, purely inhibitory responses or an initial inhibitory phase before an excitatory response appeared in addition to the excitatory responses to the pheromone (Figure 8). When stimulated with 0.1% heptanal, more than half of the neurons responded with pure inhibition (e.g., neurons 3, 5, 6, 7 in Figure 8) or not at all (e.g., neurons 2, 8, 9 in Figure 8). Also the evolution of response patterns from 0.1 to 1% heptanal was highly variable (Figure 8).

**Figure 9:**
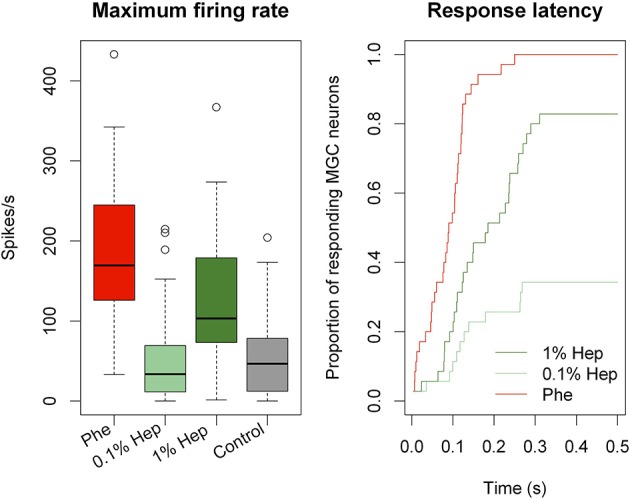
**Quantitative analysis of MGC neuron responses to the pheromone and heptanal**. Boxplot of mean maximum firing rates (Box = median with first and third quartiles, whiskers = second and fourth quartiles, circles = outliers) and Kaplan-Meier estimator curves for the latencies of MGC neuron (*n* = 35) responses. MGC neurons respond with lower maximum firing rates and longer latencies to 0.1 and 1% heptanal compared to the pheromone. Control refers to pooled data of stimulation with pure hexane and pure mineral oil. Hep, heptanal; Phe, pheromone.

To compare responses between the three stimuli quantitatively, we pooled all neurons displaying an excitatory phase and compared maximum firing rates and latencies statistically (Figure 9). Maximum firing rates in MGC neurons were significantly higher in response to the pheromone than to 0.1% heptanal (Wilcoxon signed rank test for paired data, *V* = 625, *p* = 5.821^−10^) and to 1% heptanal (*V* = 535, *p* = 1.51^−5^). Maximum firing rates in response to 1% heptanal were significantly higher than responses to control (*V* = 561, *P* = 1.454^−5^) but not to 0.1% heptanal (*V* = 295, *p* = 0.972). Response latencies were also statistically different between the three stimuli (χ^2^ = 65.2 on 2 degrees of freedom, *p* = 7.11^−15^). But latencies in response to the pheromone were not only shorter, but also less variable than in response to heptanal (Figure 9). The Kaplan-Meier estimator curves for latency illustrate the larger spreading of response latencies to heptanal, especially to the lower concentration and the large proportion of non-responding neurons (Figure 9).

### Heptanal does not trigger complete upwind flight

Male moths were generally very active in the wind tunnel, even in the absence of an olfactory stimulus as shown by the high percentage of activation (78%) and take off (56%) in control tests (Table 2). However, none of these active moths reached the upwind end of the tunnel in control experiments. A significantly higher percentage of males were taking off (96.0%, χ^2^ = 19.73 *p* < 0.001) and performed a sustained flight (92.0%, χ^2^ = 19.42 *p* < 0.001), reaching the half-length of the tunnel before the end of the test (34%, χ^2^ = 48.52 *p* < 0.001) in presence of the pheromone compared to the control stimulation. Males took off significantly earlier in response to the pheromone (median time for take off = 21.5 s), compared to heptanal 0.1 and 1% or control (59.0, 53.0, and 56.0 s, respectively; χ^2^ = 48.7 on three degrees of freedom, *p* = 1.51^−10^). Male *A. ipsilon* arrived close to the source only in presence of the pheromone. There was no statistical difference between heptanal at 0.1% and control stimulation for all items. In turn significantly more males took off (79.2%; χ^2^ = 19.73, *p* < 0.001) and performed a partial flight (75.0%; χ^2^ = 19.42, *p* < 0.001) when stimulated with heptanal at 1%, compared to controls (Table 2).

**Table 2:** **Flight responses of virgin male *A. ipsilon* to heptanal and the pheromone in a wind tunnel**.

**Stimulus**	**Number of males**	**Activation**	**Taking off**	**Partial flight**	**Source flight**
Pheromone	50	100	96.0	92.0	34.0
0.1% heptanal	26	84.6	76.9	61.5	0.0
1% heptanal	24	91.7	79.2	75.0	0.0
Control	50	78.0	56.0	50.0	0.0

Data are the percentages of four behavioral items carried out within 90 s.

**Figure 10:**
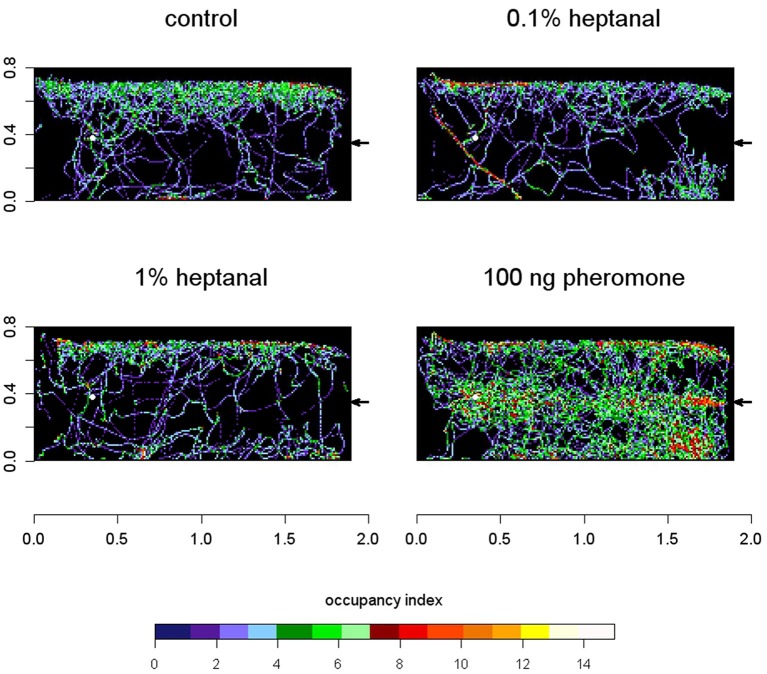
**Males perform upwind flight to pheromone but not to heptanal in a wind tunnel**. Occupancy maps present side views of the summated tracks of male *A. ipsilon* in the wind tunnel. *n* = 37 tracks (100 ng pheromone); *n* = 21 (0.1% heptanal), *n* = 18 (1% heptanal), *n* = 25 (control). The arrow at the right of each diagram indicates the position of the stimulus source and the direction of the wind. The white dot indicates the point of male departure. Scales are given in meters. To draw the map, the summated numbers of recordings of a male presence within a 1 cm square were calculated from the coordinates of 3D tracks and color-coded.

In presence of the pheromone, the distribution map of moths in the wind tunnel revealed a strong density of male presence in the longitudinal axis downwind to the pheromone source (Figure 10). In turn, in presence of both concentrations of heptanal, males did not fly upwind. Control tracks showed a preference of male moths for the front side revealing a possible un-controlled heterogeneity in the room environment but no preference for the longitudinal median axis.

## Discussion

### Phe-ORNs are sensitive to volatile plant compounds

Using complementary approaches, we show that heptanal and other plant odorants activate the pheromone-specific pathway in male *A. ipsilon*. Still, heptanal was less efficient than the sex pheromone—dose response curves were shifted toward higher concentrations—thus behaving as a partial agonist at the detection level. Although we used a three-component pheromone blend for stimulation, we suppose that we primarily investigated the detection of plant odorants within Phe-ORNs tuned to the major pheromone component Z7-12:Ac, because these ORNs represent more than 90% of all pheromone-detecting neurons on the antennal branches, which we recorded from Renou et al. (1996). Insect olfactory receptors (ORs) involved in the detection of compounds having high biological relevance to the insect's biology show generally narrow tuning ranges (Wang et al., 2010) confering high selectivity to ORNs that express them. Among ORNs, Phe-ORNs are thus considered as specialists, narrowly tuned to individual pheromone constituents and not responding to general odorants (Masson and Mustaparta, 1990). However, some exceptions to this rule are known: receptor neurons tuned to the main pheromone component, codlemone, in the antennae of male *Cydia pomonella* respond also to pear ester, ethyl (E,Z)-2,4-decadienoate (De Cristofaro et al., 2004). In this particular example, the structural resemblance between codlemone and pear ester, could account for the capacity of this compound to excite Phe-ORNs. The OR for codlemone has not yet been found but curiously, CpomOR3, later identified as a specific receptor for pear ester (Bengtsson et al., 2014) and having a close relationship with moth pheromone receptors, did not bind components of the codling moth pheromone blend. In turn, responses of Phe-ORNs to short chain alcohols or monoterpenes have been mentioned in *A. segetum* and *S. exigua* whose pheromones are long-chain acetates (Hansson et al., 1989; Dickens et al., 1993). Besides these few examples of their direct activation by VPCs, Phe-ORNs' specificity can also be challenged by mixture interactions with pheromone as reported in various moth species (Rouyar et al., 2011 and references therein). However, to our knowledge, the present work is the first detailed report of a plant odorant with no chemical similarity to the molecular structure of the pheromone acting on its own as a partial agonist of a moth pheromone. Besides heptanal several other plant volatile compounds were found to activate the firing of Phe-ORNs in *A. ipsilon*. In turn, most Phe-ORNs did not respond to α-pinene. To determine whether the aldehyde function was important for activity, we tested two other short chain aldehydes, hexanal and octanal. Both compounds did not elicit higher responses compared to the other VPCs, showing that there is no simple correlation between chemical structure and the capacity to excite Phe-ORNs. The finding that Phe-ORNs respond to higher concentrations of VPCs compared to pheromone is well in agreement with the observation that OR specificity is generally dose dependent, an increase in the concentration of the odorants broadening the response spectra (Hallem and Carlson, 2006). Although we cannot exclude that *A. ipsilon* Phe-ORNs might express multiple OR types, we hypothesize that pheromone-binding ORs are activated by high doses of heptanal in this species in analogy to what has been found for interactions between pheromones and plant odors at the molecular level: Modifications of pheromone responses by certain plant volatiles have been shown to be dependent on a pheromone-specific OR in *Heliothis virescens* (Pregitzer et al., 2012). It should be noted that in the field VPC concentrations needed to activate Phe-ORN might be reached only very close to their plant sources.

Contrasting with this ability of heptanal, and to lesser extent linalool, to activate pheromone receptors in *A. ipsilon*, both compounds did not activate the Phe-ORNs tuned to the main pheromone component in *S. littoralis*. On the contrary, linalool has been shown to be an antagonist of pheromone detection in the latter species (Party et al., 2009). Such differences between moth species support the hypothesis that activation of heptanal might not be an unspecific pharmacological effect. Differences between the two moth species might result from binding affinity in the pheromone ORs for heptanal, correlated to the different chemical structure of their pheromone ligands. Alternatively, this cross-sensitivity of *A. ipsilon* Phe-ORNs, but not those of *S. littoralis*, to pheromone and heptanal could reflect a specific adaptation of male *A. ipsilon* due to the ecological advantage for them to detect plant odorants attractive to sexually mature females (Landolt and Phillips, 1997).

### MGC neurons are sensitive to volatile plant compounds

Calcium imaging at the AL level showed strong calcium responses to heptanal, and to a lesser degree to linalool in the MGC, showing that the activation of Phe-ORNs by VPCs produces a strong input in the pheromone-specialized areas of the olfactory centers. Interestingly, the size of the activated area in the MGC and the number of activated OGs was larger at the high concentration (1%), compared to the low concentration (0.1%) suggesting a broadening of responses at high stimulus concentration, probably due to additional responses from ORNs which are less tuned to the ligand.

Almost all MGC neurons responding to 100 ng of the pheromone were also activated by 1% heptanal. However, maximum firing rates were significantly lower with 1% heptanal and the response patterns were much more variable than those to the pheromone and in many cases included an initial inhibition before the excitatory response. These results are in accordance with earlier studies in *A. ipsilon*, where both stimuli elicited responses, although tested only at lower doses (Jarriault et al., 2009; Barrozo et al., 2011; Chaffiol et al., 2012). Activation of MGC neurons by VPCs at behaviorally active doses has been also reported in several other moth species: *S. littoralis* (Anton and Hansson, 1995), *Manduca sexta* (Reisenman et al., 2008), *C. pomonella* (Trona et al., 2010), and *Cydia molesta* (Varela et al., 2011). This across-pathway stimulation has so far essentially been explained to originate from the AL network linking ordinary glomeruli to the MGC by local interneurons (LNs) and thus allowing indirect input of VPC information to the MGC via interglomerular excitation and/or inhibition and allowing interactions between different odorants at the central nervous level (Lei and Vickers, 2008). Our new data in *A. ipsilon* show now that VPC activation within the MGC is probably a combination of direct activation of Phe-ORNs by VPCs directly transmitted to the MGC and indirect activation through the network activity of local interneurons, themselves receiving VPC-ORN input within OGs. In those MGC neurons, which respond in the same way to the pheromone and heptanal, it is likely that a direct activation of Phe-ORNs by VPCs is dominant. The more variable response patterns to heptanal and specifically the initial inhibition in the heptanal responses in the remaining MGC neurons not occurring for the pheromone, indicates that input from VPC neurons might activate the inhibitory LN network of the AL. In the future we will need to test the contribution of the AL network to global MGC input and individual MGC neuron responses to volatile plant components by different experimental approaches, using for example blockers of GABA or histamine, the main local interneuron transmitters (Galizia and Szyszka, 2008).

### Behavioral significance of responses to VPCs in the pheromone subsystem

Since heptanal activated Phe-ORNs and central neurons in the MGC it was interesting to investigate the behavioral consequences. Moths' upwind flight responses to floral volatiles that signal for nectar sources are well established (Riffell et al., 2013). Male *A. ipsilon* were found to perform upwind flights not only to a linden flower extract in a wind tunnel (Barrozo et al., 2010a), but also in response to 100 μg of heptanal disposed on a filter paper (Deisig et al., 2012). Looking only at male flight scores in the wind tunnel does not reveal if an odor is perceived as a sexual or a feeding signal. We undertook here a more precise comparison of male flight behavior in the wind tunnel in presence of pheromone or heptanal with the same stimulation device as in physiological experiments. Male moths did take off in presence of heptanal and performed sustained flight at the high heptanal dose but they did not orient to the source. This absence of oriented flight toward heptanal in virgin males that responded readily to the pheromone strongly suggests that in spite of the activation of the pheromone pathway male moths did not perceive heptanal as a sexual signal. Since heptanal triggered intense activity in the MGC, the question is to determine which part of the olfactory system is responsible to operate discrimination of the pheromone from general odorants when the chemical specificity of Phe-ORNs is challenged. The convergence of a great number of ORNs on a few projection neurons and reciprocal interconnections between projection neurons through LNs do probably not facilitate the discrimination of heptanal from pheromone within the AL. However, a high degree of divergence occurs again between AL output and mushroom bodies, where a couple of hundred projection neurons make synaptic contact with high numbers of Kenyon cells (2500 in *D. melanogaster;* up to 170 000 in the honey bee; numbers provided in Galizia and Szyszka, 2008). Both intrinsic properties of Kenyon cells, such as active dendritic conductance, high action potential thresholds (Perez-Orive et al., 2002; Demmer and Kloppenburg, 2009) and postsynaptic inhibition (Papadopoulou et al., 2011) contribute to sparse coding within the mushroom bodies. This particular circuitry described in the upper levels of the olfactory system might allow better extraction of pheromone information from the contextual odorants (essentially a background of plant odors), especially when those contextual odorants trigger some activity in the pheromone sub-system. In addition, a spatially much broader representation of heptanal within the antennal lobe compared to the pheromone might contribute to the discrimination between the two signals.

### Ecological relevance of heptanal cross-activity

Although heptanal is not rare among the volatile organic compounds emitted by plants, its role in insect chemical ecology is relatively poorly documented. References in the literature indicate that this aldehyde is present in odor blends that have proven to be either attractive or repellent according to the species considered. Its production and release are induced for instance in maize following its infestation by leafhoppers, but the semiochemical activity on the insects has not been assessed (Oluwafemi et al., 2012). Heptanal has been reported to attract ovipositing females of the potato tuber moth, *Phtorimaea operculella* (Ma and Xiao, 2013). In turn, it is a component of synthetic blends designed as repellent for conifer infecting bark beetles (Huber et al., 2001). Blossoms of linden (*Tilia americana*) are intensively visited by adult *A. ipsilon* as a source of nectar (Wynne et al., 1991; Zhu et al., 1993). Blooming linden liberate huge amounts of volatiles among which heptanal is a major constituent so that it might be an environmental cue to *A. ipsilon* males indicating food sources and indirectly also the presence of females. This could explain that developing sensitivity to high concentrations of heptanal might be advantageous in the context of pheromone communication for males, largely compensating the drawback associated to reduced specificity of the pheromone sub-system. *S. littoralis*, on the other hand, is mainly distributed in Africa and the middle east. Adults feed on a large variety of flowering plants, including e.g., Solanaceae, citrus, and clover. Even though heptanal is emitted in small amounts from a variety of flowering plants visited by *S. littoralis*, such as strawberry (Klatt et al., 2013), it is not known to specifically attract this species. In turn, linden trees, emitting considerable amounts of heptantal and attracting *A. ipsilon* are native throughout the temperate northern hemisphere and not present in the natural habitat of *S. littoralis*. Such ecological differences might explain the differences in sensory physiology between the two moth species.

### Conflict of interest statement

The authors declare that the research was conducted in the absence of any commercial or financial relationships that could be construed as a potential conflict of interest.
